# Evaluation of satellite based rainfall estimates and drought monitoring linked to climate indices in Genale Dawa Basin, Ethiopia

**DOI:** 10.1038/s41598-025-29136-6

**Published:** 2025-11-23

**Authors:** Mehuba Demissie Lemma, Tadesse Tujuba Kenea, Amba Shalishe Shanka, Yoseph Arba Orke

**Affiliations:** 1 Faculty of Meteorology and Hydrology, Arba Minch University, Arba Minch, Ethiopia; 2 Ethiopian Meteorology Institute, Addis Ababa, Ethiopia; 3 Taiwan International Graduate Program (TIGP)-Earth System Science Program (ESS), Academia Sinica and National Central University, Academia Sinica, Taipei, 11529 Taiwan

**Keywords:** Satellite-Rainfall estimates, Climate indices, Drought monitoring, Genale dawa basin, Climate sciences, Hydrology, Natural hazards

## Abstract

Droughts severely impact agriculture, food security, and water resources, particularly in data scarce regions like the Genale Dawa River Basin (GDRB). This study evaluates the performance of five satellite rainfall products including CHIRPSv2.0, RFE2.0, TAMSATv3.1, PERSIANN-CDR, and ARC2 over 2001–2020 using metrics such as Mean Bias, Absolute Error (MAE), Root Mean Square Error (RMSE), Correlation Coefficient (CC), Probability of Detection (POD), False Alarm Ratio (FAR), and Critical Success Index (CSI). Furthermore, the studies examined the relationship between climate indices including the Pacific Decadal Oscillation (PDO), North Atlantic Oscillation (NAO), El Niño Southern Oscillation (ENSO), and Atlantic Multidecadal Oscillation (AMO) and drought variability. CHIRPSv2.0 demonstrated the highest accuracy, especially at Ginir (CC = 0.98) and Chewbet (CC = 0.75), and the lowest errors at Teferekella (MAE = 45, RMSE = 65). Based on a multi-criterion ranking approach, CHIRPSv2.0 was identified as the most reliable satellite-based product for daily rainfall estimation across the entire basin, exhibiting strong performance metrics (Lp = 0.433, CC = 0.84, POD = 0.85, CSI = 0.46). CHIRPSv2.0 was used to analyze drought spatiotemporal patterns using SPI-3 and SPI-6. Drought severity, frequency, and duration were highest in the west-central and northern GDRB, with major events occurring in 1988, 1991–1993, 2000, 2004, 2011, and 2012. Climate index analysis showed that AMO and NAO positive phases were associated with wetter conditions, while negative PDO and ENSO phases corresponded with drier periods, especially in central and eastern areas. These findings highlight CHIRPSv2.0’s reliability for drought monitoring and its value for early warning and mitigation planning in the GDRB.

## Introduction

 Drought is generally defined as a prolonged of abnormally low rainfall, leading to a shortage of water. It is one of the leading destructive natural hazards adversely affecting natural resources and livelihoods^1^ and it can occur anywhere in the world. Droughts are commonly classified into meteorological, hydrological, agricultural, and socioeconomic^2^. Meteorological drought refers to rainfall deficit compared to the long-term mean^3^ whereas hydrological drought occurs when stream flows are insufficient to meet water needs, emphasizing the need for effective water management^4^. Agricultural drought arises when soil moisture is insufficient for crop needs^5^. Socioeconomic drought results when water shortages lead to a demand for economic goods that exceeds supply^6^. Droughts affect an estimated 55 million people worldwide yearly, posing the most serious threat to animals and crops in almost every region^7^.

Developing countries are particularly vulnerable to climate change and drought due to the weak economic situation^8^. Droughts are particularly common in East African countries due to large spatial and temporal variability in rainfall^9^. Ethiopia is one of the country’s most vulnerable to climate shocks, particularly drought, due to its irregular rainfall pattern^10^. Recent studies have highlighted that Ethiopia has experienced frequent and recurrent droughts, with severe drought events occurring in most parts of the country in 2009, 2011, 2012, 2014, and 2015^11–14^.

The GDRB is one of Ethiopia’s major river basins, playing a crucial role in the country’s water resources and agricultural activities. However, the basin has been increasingly vulnerable to the impacts of climate variability, particularly in the form of frequent and prolonged drought^15,16^. These droughts not only strain the water supply for agriculture, which is the backbone of the region’s economy, but also lead to severe consequences for food security, livelihoods, and the overall socio-economic stability of the area. Reliable drought monitoring requires long-term, consistent rainfall and other climatic datasets^17^. Precipitation is an important part of the hydrological cycle that is commonly utilized to monitor and forecast droughts^18^. The measurement of rainfall is important for a variety of scientific and social applications across the globe. A meteorological drought monitoring system is heavily dependent on the availability of rainfall data at the appropriate spatial and temporal scales. Understanding the occurrence, amount, distribution, and changes in rainfall is crucial for improving meteorological drought monitoring and characterization^19^. In general, rainfall measurements from ground observation are the principal sources and provide accurate point measurements of precipitation. However ground observations precipitation data are limited in many drought-prone areas, particularly in Africa, where such records are often of poor quality and hard to access due to regulatory constraints, limited dissemination, and high costs^20,21^.

In recent decades, numerous quasi-global satellite precipitation products have been developed for hydro-meteorological applications^22^. Notable examples include TRMM Multi-satellite Precipitation Analysis (TMPA), the Climate Prediction Center Morphing Technique (CMORPH) satellite-based rainfall product, African Rainfall Climatology (ARC), Tropical Applications of Meteorology using Satellite (TAMSAT)and Precipitation Estimation from Remotely Sensed Information using Artificial Neural Networks (PERSIAN). These products offer accurate precipitation estimates with high spatiotemporal resolution and perform well in drought and other natural hazard assessments^23^.

Numerous studies across various watersheds have attempted to compare and assess satellite rainfall products with ground data^12,24–26^. In this study, authors used different types of satellite rainfall estimation such as CHIRPS, PERSIANN, CMORP, and TMPA. For example^27^, showed that some satellite precipitation products performed reasonably well on 10-day to monthly time scales, with a resolution of 2.5° over the complex topography of East Africa^25^ reported that CHIRPS has outperformed slightly and can optionally be used for meteorological drought monitoring up to developing early warning systems in the Abaya Chamo basin. In addition, ^16^ found that CHIRPSv2.0 and IMERG6 products could be reliable rainfall data sources for the hydrological analysis of the Dhidhessa River Basin compared to TAMSAT. Moreover, the performance of satellite precipitation products has been reported to vary among different regions^28^.

**Fig. 1:**
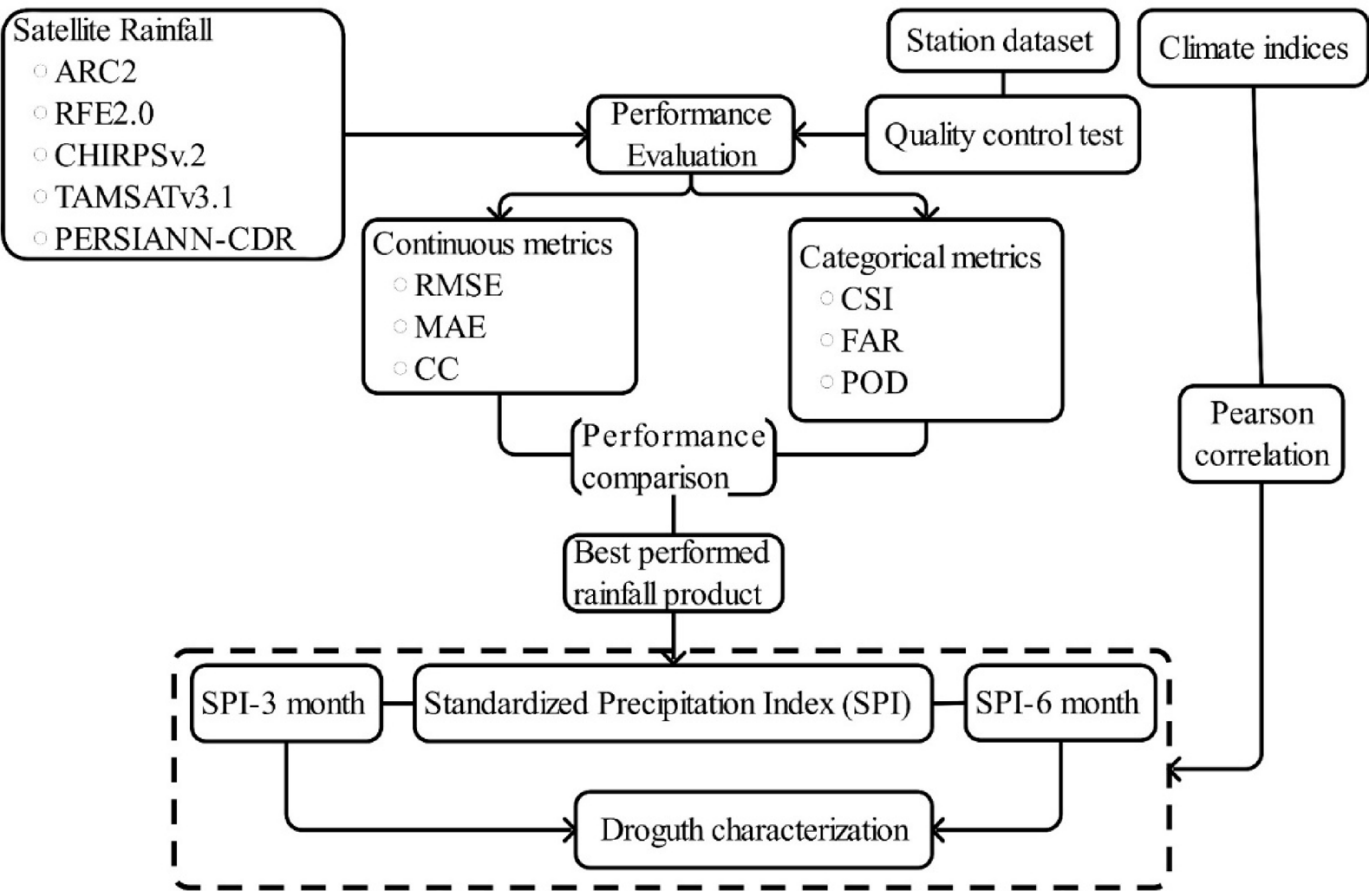
Framework of the study.

In the GDRB, the sparse distribution of rain gauges leads to unreliable rainfall data. As shown in Fig. 1, meteorological stations are particularly scarce in the southern region, with no stations in the lowland areas. This makes it difficult to accurately assess meteorological drought and address water management needs. To fill this gap, satellite-based rainfall products, offering extensive spatial and temporal coverage, should be utilized. However, these products require careful evaluation due to uncertainties arising from measurement errors, sampling biases, retrieval algorithms, and correction methods, all of which can be influenced by local topography and climate^29,30^. Therefore, accurate drought monitoring and tracking are critical in the GDRB for effective water resource management, agriculture, and disaster preparedness, ensuring sustainable livelihoods and resilience to climate variability.

Therefore, the objectives of this study are to (1) evaluate the performance of CHIRPSv2.0, RFE2.0, TAMSATv3.1, PERSIANN-CDR, and ARC2; (2) assess spatiotemporal meteorological droughts using SPI based on the best-performing satellite rainfall products; and (3) examine the association between meteorological droughts and climate indices. This study is crucial as it enhances the accuracy of drought monitoring in the GDRB, where ground observations are sparse and unreliable. By evaluating the performance of various satellite rainfall products and their correlation with climate indices, the research provides valuable insights for better understanding drought patterns and their climatic drivers. These findings will contribute to improved water resource management, agricultural planning, and disaster preparedness in a region vulnerable to drought, ultimately supporting more resilient and informed decision-making.

## Materials and methods

In this study, various satellite rainfall products were evaluated using continuous metrics and categorical metrics against quality-controlled station datasets. The best-performing rainfall product was identified and used to compute the Standardized Precipitation Index (SPI) at 3- and 6-month scales. Drought characteristics, including magnitude, duration, and frequency, were analyzed based on the SPI. Additionally, correlations between climate drivers to droughts were conducted to assess the influence of climate drivers on dry events. The flow of the study is presented in Fig. 1.

### Description of the study area

**Fig. 2:**
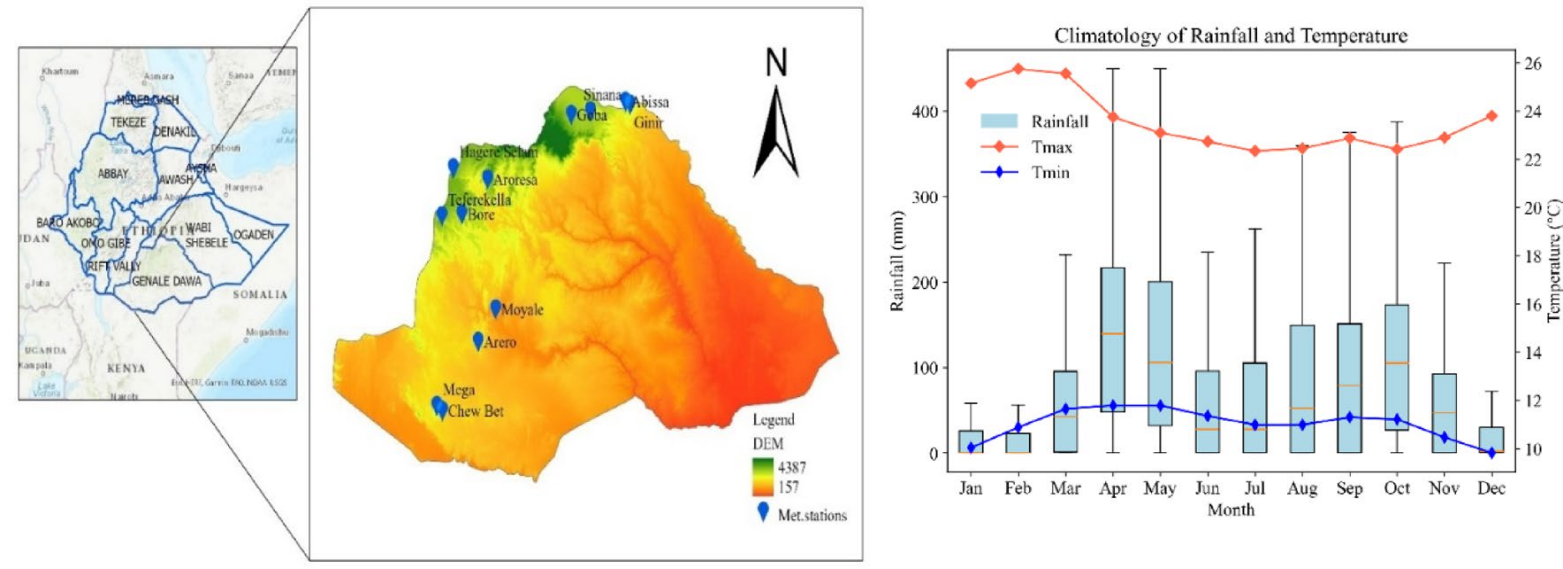
Study area map with meteorological stations, climatology of rainfall and temperature (2001–2020).

The study was conducted in GDRB. GDRB is in the southeastern region of the country, within 3.5^o^ to 7.3^o^ N and 37^o^ to 43.3^o^ E with a land area of 172,889 km^2^ (Figure 2). It is the third-largest river basin, after the Abay River and Wabi-Shebelle basins of Ethiopia^31^. The basin is also characterized by diverse topography that includes highlands, lowlands, and extensive rift valley landscape. It is characterized by diverse climatic zones, ranging from cool highlands in the north to hot lowlands in the southeast. The basin’s elevation varies from 4387 m above sea level in the highlands to 157 m at its outlet, with a general southeastward inclination (Figure 2). The climate of the basin is mainly controlled by the seasonal migration of the Inter-tropical Convergence Zone (ITCZ), which is conditioned by the convergence of trade winds of the northern and southern hemispheres and the associated atmospheric circulation. The GDRB experiences three distinct rainy seasons: two long rainy seasons, Belg (March to May) and the Autumn rains (September to October), and one short rainy season, Kiremt (June to August). This seasonal distribution of rainfall plays a crucial role in the region’s agricultural activities and water resource management. The region experiences significant climatic variability, with mean annual rainfall ranging from 1,500 millimeters in the northern highlands to as low as 200 millimeters in the southern lowlands. Temperatures also vary widely, averaging 15.5 °C in the northern highlands and 28 °C in the southern lowlands. This variability influences the basin’s hydrological processes, making it one of Ethiopia’s most drought-prone regions^32^.

### Datasets and sources

#### Observed data

Historical daily rainfall data from 2001 to 2020 were obtained from 12 meteorological stations of the Ethiopian Meteorology Institute (Table 1). These datasets were used as a benchmark to evaluate and validate satellite precipitation estimates. Data quality was assessed using the Climate Data Tool^33^ with missing values addressed through arithmetic mean and normal ratio^34^. According to WMO, stations with more than 25% missing data were excluded from the analysis to maintain reliability. However, the final selection of stations in this study was based on their superior data availability, agro-climatic representation, and temporal consistency which reduces the risk of biases associated with missing data and ensures the reliability of our analysis. Consequently, only the stations highlighted in Table 1 were used for the performance evaluation. These stations also provide a geographically representative sample of the study area, allowing for a more accurate assessment of regional climate patterns.

**Table 1:** Geographical coordinates, elevation, and mean rainfall of the station used for this study.

Stations	Latitude (*N*)	Longitude (E)	Elevation (mm)	Missed data (%)	Annual Rainfall
Abissa	7.16	40.65	1944	25	570
**Ginir**	**7.13**	**40.71**	**1941**	**7.4**	**1100**
Arero	4.72	38.83	1785	25	385
Aroresa	6.37	38.95	2504	25	1471
Bore	6.02	38.63	2712	11.3	1462
**Chew Bet**	**4.02**	**38.39**	**1472**	**9.7**	**405**
Goba	7.03	39.98	2614	13.1	601
Sinana	7.07	40.22	2400	10.4	797
H/Selam	6.49	38.52	2809	18.45	1187
Mega	4.07	38.32	1820	25	465
**Moyale**	**5.05**	**39.05**	**1166**	**5.3**	**551**
**Tefere-kella**	**6.00**	**38.38**	**1870**	**4.97**	**1799**

#### Satellite data

The study evaluated five satellite rainfall products: CHIRPSv2.0, RFE 2.0, TAMSATv3.1, PERSIANN-CDR, and ARC2. These products were chosen based on their data availability, long time series, high spatial and temporal resolutions, and accessibility for the GDRB. This study analyzed the performance of various satellite-derived rainfall datasets, which are summarized in Table 2. It includes the dataset as well as its data source, period of record, and temporal and spatial scale. These datasets provide comprehensive coverage and have been widely used in rainfall estimation studies. The reader is referred to relevant publications for more details. Their performance was evaluated in this study to assess accuracy and reliability in different climatic regions in the GDRB.

**Table 2:** Descriptions of the satellite rainfall products used in the study.

Satellite product	Spatial coverage	Spatial resolution	Temporal coverage	Temporal resolution
CHIRPS v2.0	50°N – 50°S, 0° – 360°E (Globe)	0.05°	1981 - present	Daily
RFE2	40°N – 40°S, 20°W – 55°E (Africa)	0.1°	2001 - present	Daily
TAMSAT v3.1	40°N – 40°S, 20°W – 55°E (Africa)	0.0375°	1983 - present	Daily
PERSIAN-CDR	60°N – 60°S, 0° – 360°E (Globe)	0.25°	1998 - present	Daily
ARC v2.0	40°N – 40°S, 20°W – 55°E (Africa)	0.1°	1981 − present	Daily

#### Climate indices

Climate indices are diagnostic quantities used to understand the climate system. Drought is significantly influenced by various climate indices^35^. This study evaluates the association between meteorological drought in GDRB and climate indices such as the Atlantic Multidecadal Oscillation (AMO), Pacific Decadal Oscillation (PDO), North Atlantic Oscillation (NAO), and El Niño-Southern Oscillation (ENSO). These indices were selected due to their known impact on precipitation patterns and drought occurrence^36–38^. The AMO, PDO, and NAO are closely related to regional climate variability, such as temperature and precipitation fluctuations, while ENSO is particularly influential in driving anomalous rainfall and temperature patterns that contribute to both short-term and long-term drought conditions. By examining the relationship between these indices and drought events, this study aims to explore how large-scale climate drivers influence local drought patterns in the GDRB. The climate indices data were sourced from the National Oceanic and Atmospheric Administration’s (NOAA) Earth Systems Research Laboratory (ESRL), accessible at https://psl.noaa.gov/data/climateindices/list/.

### Methods

#### Satellite performance evaluation

Satellite rainfall estimates were evaluated using categorical metrics^39^ like probability of detection (POD), false-alarm ratio (FAR), and critical success index (CSI), along with continuous error metrics^40^ such as mean absolute error (MAE), root mean square error (RMSE), and coefficient of determination (R²). POD measures the fraction of observed events that were correctly detected by the satellite, while FAR quantifies the fraction of detected events that were false alarms. CSI is used to visualize the fraction of data points that are correctly estimated to have values above the threshold. These metrics ranging from − 1 to + 1, indicate how well the satellite product rainfall reflects the temporal variability detected in the observed data, with values closer to + 1 signifying a strong positive correlation.

RMSE was employed to quantify the magnitude of errors between the satellite-derived data and observed data. It is particularly useful when comparing the performance of different satellite datasets. MAE was used to assess the average absolute difference between satellite-derived and observed data. It is less sensitive to outliers compared to RMSE, offering a straightforward interpretation of the average error magnitude, which is important for understanding the general performance of the satellite data. Detailed explanations and formulas for these metrics are provided in Table 3 and the algorithm for categorical performance indices^41^ is provided in Table 4.

**Table 3:** List of statistical indicators used in the evaluation process.

Statistical indicators	Formula	Perfect Score
Continuous statistics		
Coefficient of determination	$\:{\mathrm{R}}^{2}={\left(\frac{\sum\:_{\mathrm{i}=1}^{\mathrm{n}}{\left[\right(\mathrm{X}}_{\mathrm{o}\mathrm{i}-}\stackrel{-}{{\mathrm{X}}_{\mathrm{o}}\:}\left)\right({\mathrm{X}}_{\mathrm{s}\mathrm{i}}-\stackrel{-}{{\mathrm{X}}_{\mathrm{s}}\:}\left)\right]}{\sqrt{\sum\:{{(\mathrm{X}}_{\mathrm{o}\mathrm{i}-}\stackrel{-}{{\mathrm{X}}_{\mathrm{o}}\:})}^{2}\mathrm{*}{({\mathrm{X}}_{\mathrm{s}\:\mathrm{i}}-\stackrel{-}{{\mathrm{X}}_{s}\:})}^{2}}}\right)}^{2}$	1
Relative Bias	$\:\mathrm{R}\mathrm{B}=\frac{1}{\mathrm{n}}\sum\:_{\mathrm{i}=1}^{\mathrm{n}}{(\mathrm{X}}_{\mathrm{s}\:\mathrm{i}}-\:\:{\mathrm{X}}_{\mathrm{o}\mathrm{i}}\:)$	1
Mean Absolute Error	$\:\mathrm{M}\mathrm{A}\mathrm{E}=\frac{\frac{1}{\mathrm{n}}\sum\:\left|\left(\mathrm{X}\mathrm{o}\mathrm{i}-{\mathrm{X}}_{si}\right)\right|}{{X}_{s}}$	0
Root Mean Square Error	$\:RMSE=\sqrt{\frac{1}{n}\sum\:_{i=1}^{n}{({X}_{oi}-{X}_{si})}^{2}}$	0
Categorical statistics		
Probability of Detection	$\:POD=\frac{H}{(H+M)}$	1
Critical Success Index	$\:\mathrm{C}\mathrm{S}\mathrm{I}=\frac{\mathrm{H}}{(\mathrm{M}+\mathrm{F}\mathrm{A}+\mathrm{H})}$	1
False Alarm Ratio	$\:\mathrm{F}\mathrm{A}\mathrm{R}=\frac{\mathrm{f}\mathrm{a}\mathrm{l}\mathrm{s}\mathrm{e}\:\mathrm{a}\mathrm{l}\mathrm{a}\mathrm{r}\mathrm{m}\mathrm{s}}{\mathrm{h}\mathrm{i}\mathrm{t}\mathrm{s}+\mathrm{f}\mathrm{a}\mathrm{l}\mathrm{s}\mathrm{e}\:\mathrm{a}\mathrm{l}\mathrm{a}\mathrm{r}\mathrm{m}\mathrm{s}}$	0

**Table 4:** Algorithm for categorical performance indices.

Observed/Satellite	Above threshold	Below threshold
Above threshold	Hits (H)	False Alarm (F)
Below threshold	Miss (M)	Correct Negative (CN)

Where $\:{X}_{si}$ is the $\:{\mathrm{i}}^{\mathrm{t}\mathrm{h}}\:$ value from the satellite, and $\:{X}_{obsi}$ represents the $\:{i}^{th}$observed value at the weather station, and n is the total number of data points, with $\:i\:$being the index. $\:\stackrel{-}{{X}_{o}}$ and $\:\stackrel{-}{{S}_{o}}$ are the average precipitation values for the observed and satellite data, respectively. Additionally, $\:H$ represents the number of hits,$\:\:M$ the number of misses, $\:FA$ the number of false alarms, and $\:CN$ the correct negatives.

#### Ranking of satellite rainfall Estimation

To evaluate and rank satellite rainfall estimation (SRE) products, this study employed an entropy based multi-criteria decision-making framework^42^. The method assigns objective weights to each performance indicator based on its information content, thereby reducing subjectivity and ensuring a balanced assessment^43,44^. Initially, all performance indicators were normalized to the range [0, 1] using min-max normalization to eliminate unit and scale differences. The entropy ($\:{H}_{j})$ value for each indicator j was then calculated as:

1 $$\:{H}_{j}=-\frac{1}{\mathrm{ln}\left(n\right)}\sum\:_{i=1}^{n}{k}_{ij}lnln({k}_{aj})\:for\:j=\mathrm{1,2},\dots\:.N$$

2 $$\:{k}_{aj}=\frac{{k}_{j}}{\sum\:_{i=1}^{n}{k}_{j}\left(i\right)}$$

3 $$\:{D}_{j}=1-{H}_{j}$$

4 $$\:{W}_{j}=\frac{{D}_{j}}{\sum\:_{j=1}^{N}{D}_{j}}$$

5 $$\:{L}_{p}={\left[\sum\:_{j=1}^{N}{{w}_{j}\left|{{f}_{j}}^{*}-{f}_{j}\left(i\right)\right|}^{p}\right]}^{\frac{1}{p}}$$

Where $\:{H}_{j}$ represent the entropy of each performance indicator $\:j$; $\:n$ is the total number of SRE datasets; $\:{k}_{j}\left(i\right)$ denote the value of indicator $\:j$ for the $\:i$^th^ SRE; $\:N$ represents the total number of performance indicators used. $\:{D}_{j}$ refers to the degree of diversification for indicator $\:j$, and $\:{W}_{j}$ denotes the normalized weight assigned to indicator $\:j$. $\:{L}_{p}$ represents the metric used to evaluate SRE, based on the chosen distance parameter $\:p$. The term $\:{f}_{j}\left(i\right)$ is normalized value of indicator $\:j$ for estimation $\:i$; while $\:{{f}_{j}}^{*}$ denotes the ideal (normalized) value for indicator $\:j$; $\:P$ is distance parameter [$\:p=1$ for linear distance and $\:p=2$ for squared Euclidian distance].

#### Standardized precipitation index (SPI)

SPI is widely recognized as one of the most reliable and commonly used tools for characterizing drought events globally^45^. It facilitates early drought warning and provides a quantitative assessment of drought severity based solely on rainfall data, making it a critical resource for effective drought monitoring and management. The SPI can be calculated for any period of interest, typically over intervals of 1, 3, 6, 9, 12, or 24 months. These time scales are appropriate for tracking various droughts (e.g., meteorological, agricultural, and hydrological). The SPI is spatially invariant^46,47^ therefore values of the SPI can readily be compared across time and space.

In this study, the SPI was calculated for 3-month and 6-month time scales to assess drought conditions. The severity of drought is categorized according to the SPI values, as presented in Table 5. These categories help in understanding the intensity and extent of drought in the region. The frequency distribution of cumulated rainfall is first fitted with an appropriate probability density distribution, which is then converted to a standard normal distribution. The gamma distribution (two parametric) is employed because it fits rainfall data better^48^. SPI is calculated by fitting a gamma probability density function to a given frequency distribution of rainfall totals in both pixel-based and point-based cases. Based on the work of^49^ the gamma function is mathematically expressed as follows:

6 $$\:g\left(x\right)=\frac{1}{{\upbeta\:}\varGamma\:\left(\alpha\:\right)}{X}^{{\upalpha\:}-1}{e}^{\raisebox{1ex}{$-X$}\!\left/\:\!\raisebox{-1ex}{$\beta\:$}\right.}\:\:\:,\:for\:\:x>o,$$

Where α > 0 is a shape parameter; β > 0 is a scale parameter; x > 0 is the rainfall amount, and $\:\varGamma\:\left(\alpha\:\right)={\int\:}_{0}^{\propto\:}{y}^{\alpha\:-1}{e}^{-y}dy$ is the gamma function.

7 $$\:{SPI}_{ij}=\frac{{X}_{ij-}{\mu\:}_{ij}}{{\sigma\:}_{ij}}$$

Where $\:{\mathrm{S}\mathrm{P}\mathrm{I}}_{\mathrm{i}\mathrm{j}}$ is the SPI of the $\:{i}^{th}$ month at the $\:{j}^{th}$ timescale, $\:{X}_{ij}$ is the rainfall total for the $\:{i}^{th}$ month at the $\:{\mathrm{j}}^{\mathrm{t}\mathrm{h}}$ time scale, and $\:{{\upmu\:}}_{\mathrm{i}\mathrm{j}}$ and $\:{{\upsigma\:}}_{\mathrm{i}\mathrm{j}}$ are the long-term mean and standard deviation associated with the $\:{\mathrm{i}}^{\mathrm{t}\mathrm{h}}$ month at the $\:{\mathrm{j}}^{\mathrm{t}\mathrm{h}}$ time scale, respectively.

**Table 5:** Classification of the severity of drought events on the calculation of SPI^50^.

Index Value	Class SPI	Value	Drought severity class
SPI ≥ 2.0	Extremely wet		
SPI 1.5 ≤ SPI < 2.0	Very wet	Above 0	No drought
1.0 SPI ≤ 1.5	Moderately wet	-	-
−1.0 < SPI < 1.0	Nearly Normal	−0.99 to 0	Slight drought
−2.0 < SPI≤−1.5	Severely dry	−1.99 to −1.5	Severe drought
SPI≤−2.0	Extremely dry	−2 and less	Extreme drought

#### Evaluating drought characteristics

Drought characteristics can be evaluated by the following essential features: duration, frequency, intensity, severity, and spatial and temporal extent^51,52^. In this study, drought occurrences were evaluated based only on months with SPI values corresponding to moderate, severe, and extreme droughts, as mild drought values slightly deviate from normal conditions. The characterization of drought events in terms of duration, magnitude, and frequency was carried out as follows:

The frequency of drought (F), expressed as a percentage, is calculated by dividing the number of months (n) where the SPI value meets a predetermined threshold (SPI ≤ − 1) by the total number of months in the series (N), and then multiplying by 100^48^.

8 $$\:F=\frac{n}{N}\times\:100$$

Duration (D) is the length of a drought episode and is calculated as the cumulative sum of the index value based on the duration of drought occurrence^53^.

9 $$\:D=\sum\:_{i=1}^{n}{d}_{i}$$

Drought magnitude (M) is calculated as the sum of the drought index values (Index) over the entire duration of the drought, expressed mathematically as:

10 $$\:M=\sum\:_{i=1}^{D}Index$$

#### Analysis of the association between drought and climate indices

The association between drought and climate drivers such as ENSO, AMO, NAO, and PDO was assessed using the Pearson correlation coefficient. Correlation provides a measure of the statistical relationship between variables, indicating whether changes in the magnitude of one variable are associated with changes in another, either in the same direction (positive correlation) or in the opposite direction (negative correlation). In this study, we used Pearson correlation instead of more complex methods like wavelet coherence analysis because our dataset is relatively short. Wavelet coherence needs a long dataset to give reliable results over time and different frequencies analyses^54,55^. Given the limited data, Pearson correlation was a more suitable and reliable choice. It offers a simple yet effective way to measure the strength and direction of the relationship between drought indices and climate drivers within the constraints of the available data.

11 $$\:{r}_{xy}=\frac{{\sum\:}_{i=1}^{n}\left({x}_{i}-\stackrel{-}{x}\right)\left({y}_{i}-\stackrel{-}{y}\right)}{\sqrt{{\sum\:}_{i=1}^{n}{\left({x}_{i}-\stackrel{-}{x}\right)}^{2}{\sum\:}_{i=1}^{n}{\left({y}_{i}-\stackrel{-}{y}\right)}^{2}}}$$

Where: $\:{r}_{xy}$: Pearson correlation coefficient between variables $\:x$ and $\:y$; $\:n$: number of observations; $\:xi$ : the value of $\:x$ for the $\:{i}^{th}$observation; $\:{y}_{i}$: the value of $\:y$ for the $\:{i}^{th}$ observations; $\:\stackrel{-}{x}$: mean of $\:x$ values and $\:\stackrel{-}{y}$: mean of $\:y\:$values.

## Results

### Performance evaluation of satellite rainfall estimates

#### Daily comparison of Multi-Satellite rainfall estimate

**Fig. 3:**
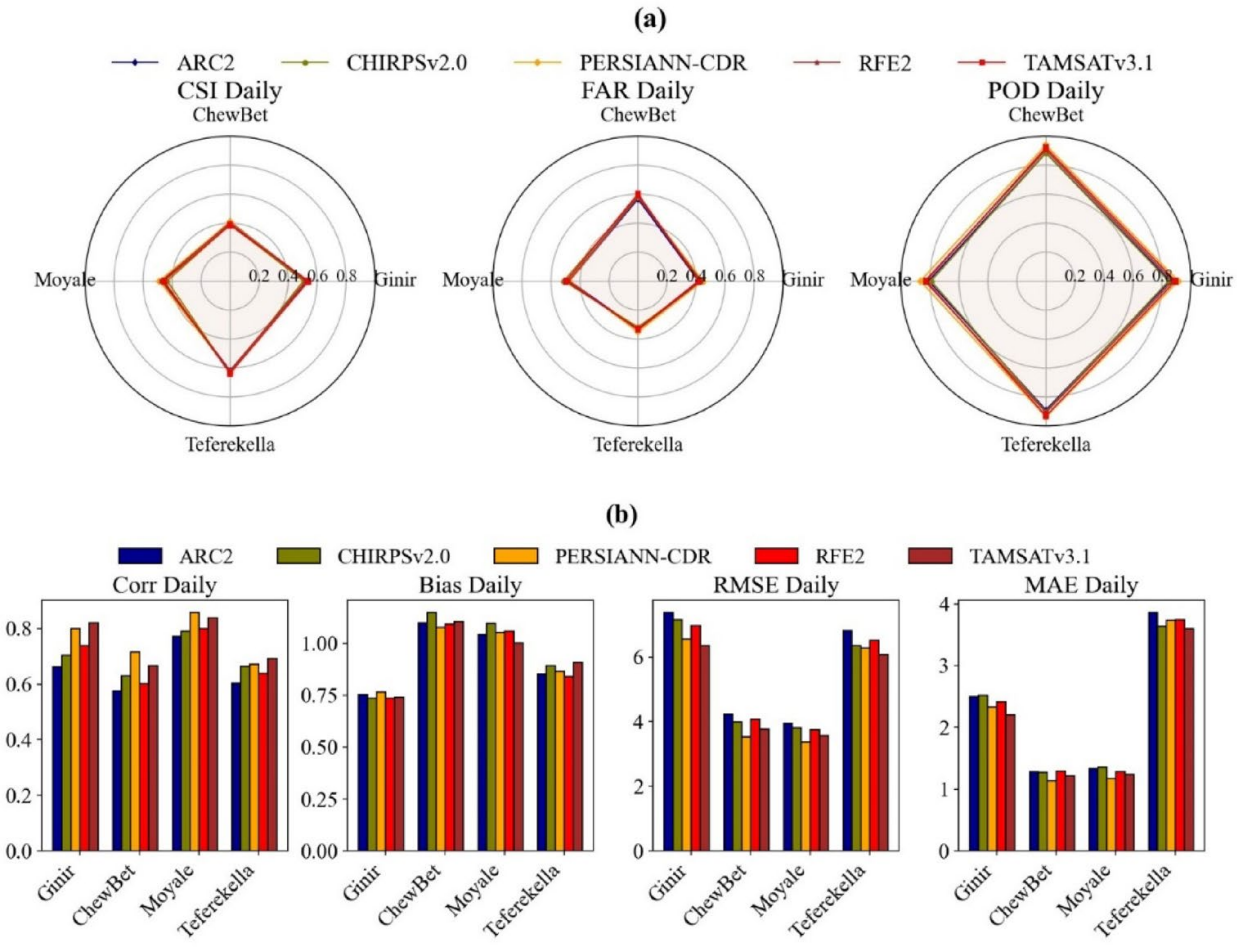
Continuous statistical evaluation (**a**) and categorical metric (**b**) performance of various satellite products at each station on a daily timescale.

Figure 3 shows the performance evaluation of different satellite datasets for four stations based on categorical (a) and continuous (b) metrics for daily time scales. As per the result, the values of CSI range from 0.5 to 0.65 at Ginir and Teferekella stations for all satellite products whereas at the Chewbet and Moyale stations, the values range from 0.35 to 0.45. However, the CSI value of TAMSATv3.1 is high at the Ginir (0.54) and Teferekella (0.64) stations whereas PERSIANN-CDR performed well for the Chewbet (0.41) and Moyale (0.49) stations. This indicates that TAMSATv3.1 and PERSIAN-CDR are the best-performing products for accurately measuring events across different geographical regions. The low values of FAR < 0.35, were observed at the Teferekella stations, indicating better performance. Conversely, higher FAR values > 0.4, were recorded at the other stations. The best FAR performance was at the Teferekella stations, with CHIRPSV2.0 (0.3) and TAMSAT v3.1 (0.33) performing particularly well. In contrast, the poorest performance was observed at the Chewbet and Moyale stations, with FAR values ranging from 0.46 to 0.6, where ARC2 and PERSIANN-CDR showed the best performance. As for the CSI, PERSIANN-CDR outperformed other satellite products across all stations, with the values ranging from 0.86 at Moyale to 0.94 at Teferekella on daily timescales.

The correlation coefficient for daily time scales ranges from 0.55 to 0.85 for all satellites. TAMSATv3.1 had the highest correlation coefficients, with *r* = 0.83 at Ginir and 0.7 at Teferekella stations, while PERSIANN-CDR showed the highest correlations at Chewbet and Moyale stations, with coefficients of 0.73 and 0.85, respectively. In addition, TAMSATv3.1 and PERSIANN-CDR showed lower ME and RMSE values than other products, demonstrating superior performance across all stations. However, TAMSATv3.1 performed better at Ginir and Teferekella stations, while PERSIANN-CDR excelled at Chewbet and Moyale stations for both MAE and RMSE. Overall, TAMSAT and CHIRPSV2.0 demonstrated superior performance in high-altitude regions, while PERSIANN-CDR excelled in the low-altitudes compared to other products in both categorical and continuous analyses at daily time scales.

#### Dekadal and monthly comparison of Multi-Satellite rainfall estimate

**Fig. 4:**
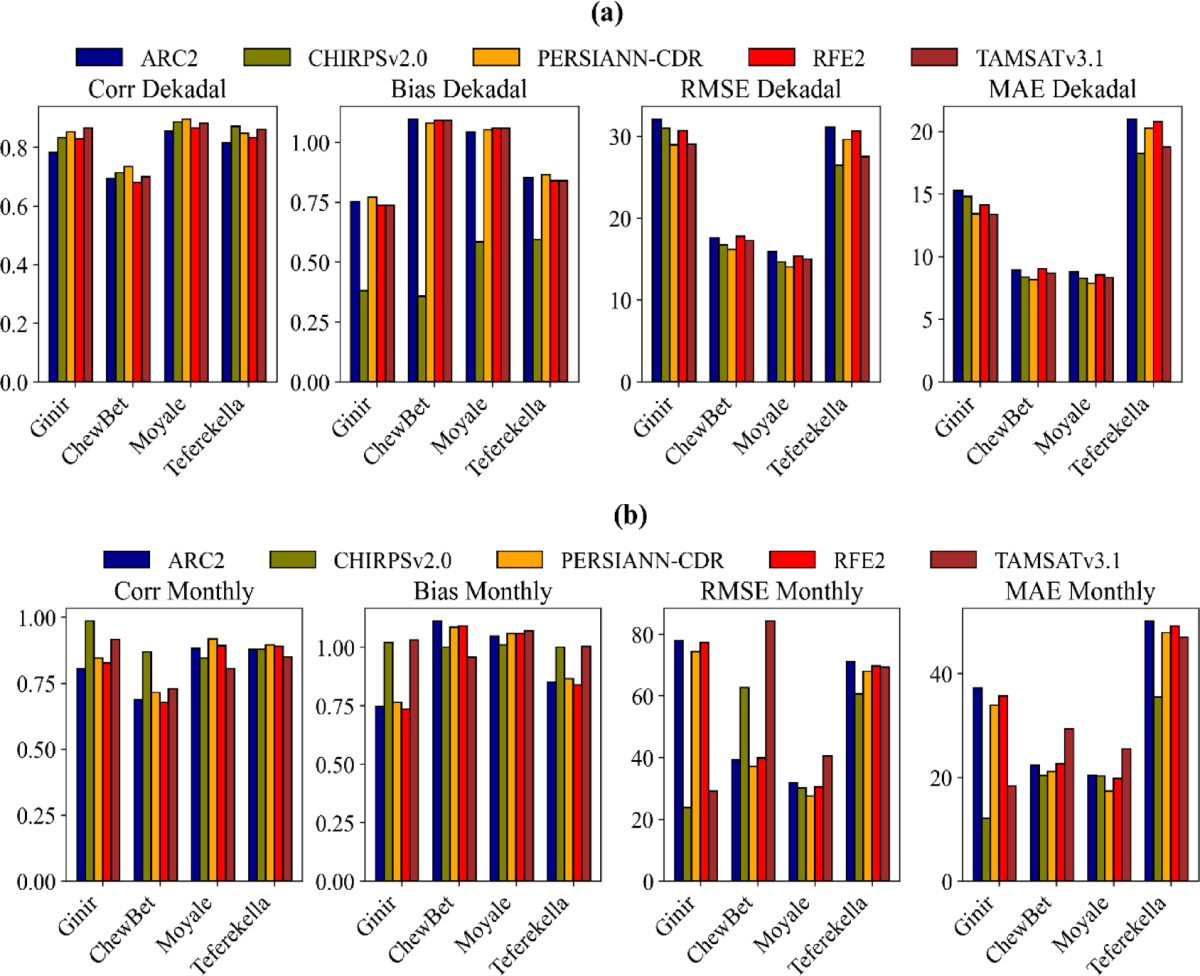
Continuous metric performance of various satellite products at each station on a dekadal (**a**) and monthly (**b**) timescale.

Figure 4 illustrates the performance evaluation of various satellite products for four stations based on continuous metrics for dekadal (a) and monthly (b) time scales. At the dekadal scale, the correlation coefficient values range from 0.65 at Chewbet to 0.89 at Moyale station. PERSIANN-CDR performed best at Chewbet and Moyale stations, followed by CHIRPv2, while CHIRPS excelled at Teferekella and TAMSATv3.1 at Ginir station. Low RMSE (13.5) and MAE (7.1%) values, indicating good performance, were observed at Chewbet and Moyale stations. Conversely, higher RMSE (32.1) and MAE (20.5) values were recorded at Ginir and Teferekella stations. PERSIANN-CDR performed best at all stations except Teferekella, where CHIRPS showed superior performance. This indicates that different products perform optimally at different locations, indicating the importance of site-specific validation when selecting rainfall estimation products.

At monthly time scales, the correlation coefficient ranges from 0.67 at Chewbet station to 0.93 at Ginir station. CHIRPSv2 performed best at Ginir (CC = 0.98) and Chewbet (CC = 0.75), followed by TAMSATv3.1. PERSIANN-CDR performed best at Moyale (CC = 0.91) and Teferekella (CC = 0.88), followed by RFE2. CHIRPSv2.0 demonstrated superior accuracy with minimal errors at Ginir (MAE = 16, RMSE = 26) and Teferekella (MAE = 45, RMSE = 65) stations. Similarly, PERSIANN-CDR achieved low error metrics at Chewbet (MAE = 20.1, RMSE = 37.1) and Moyale (MAE = 17.0, RMSE = 26.1) stations. This indicates that CHIRPSv2.0 and PERSIANN-CDR are highly accurate in estimating precipitation at the specified stations, as evidenced by their low MAE and RMSE values. These metrics reflect the degree to which the estimated precipitation values deviate from the observed values, with lower values indicating better performance. CHIRPSV2.0 and PERSIANN-CDR both exhibit strong performance in precipitation estimation, with CHIRPS V2.0 showing exceptional accuracy at Ginir and Teferekella, and PERSIANN-CDR excelling at Chewbet and Moyale. The high correlation coefficients at these stations indicate the reliable performance of these datasets, making them valuable for precipitation analysis. However, CHIRPSV2.0 provides more accurate and consistent precipitation estimates than PERSIANN-CDR, with lower error metrics and stronger correlation coefficients at key stations like Ginir and Teferekella, it does not provide coverage for all stations and not for all metrics. Therefore, to identify the best-performing satellite product across the entire Gelana Dawa River Basin, it is essential to evaluate spatial average performance over the basin rather than relying solely on individual station and individual metrics.

### SRE ranking based on spatially averaged performance across the GDR basin

To address spatial inconsistencies observed in station-based evaluations (Figs. 3 and 4), a basin-averaged assessment was conducted to identify the most reliable satellite rainfall estimate (SRE) for drought analysis across the GDR Basin. This evaluation applied a multi-criterion ranking approach using an aggregated performance index (Lp score), which integrates both continuous metrics (CC, MAE, RMSE) and categorical indicators (POD, FAR, CSI), as shown in Table 6.

Among the five evaluated products, CHIRPSV2.0 demonstrated the most balanced and consistent performance, achieving the lowest Lp score (0.433) and ranking first. It recorded the best correlation with observed data (CC = 0.84), low errors (MAE = 2.38; RMSE = 5.25), and strong detection ability (POD = 0.85; CSI = 0.46; FAR = 0.41). These results indicate CHIRPSV2.0 closely replicates rainfall variability and drought-relevant events across the basin. TAMSAT ranked second (Lp = 0.459), exhibiting excellent error performance (lowest MAE and RMSE) but slightly lower detection skill. PERSIANN-CDR and RFE2 followed, while ARC2 ranked last (Lp = 0.791), reflecting weaker agreement with observed rainfall patterns.

Based on these findings, CHIRPSV2.0 was selected as the primary SRE for subsequent drought analysis in this study, due to its robust performance across multiple criteria and its ability to capture both rainfall magnitude and drought events reliably across the GDRB.

**Table 6:** Ranking of satellite-based rainfall products on a daily time scale, based on continuous (, CC, BIAS, MAE, RMSE) and categorical (POD, FAR, CSI) performance indicators. An aggregated performance index (Lp​ score) was employed to integrate all these metrics and provide an objective ranking of each product. Lower Lp scores indicate better overall performance.

SRE	CC	BIAS	MAE	RMSE	POD	CSI	FAR	Lp_score	Rank
CHIRPSV2.0	0.84	1.14	2.38	5.25	0.85	0.46	0.41	0.433	1
TAMSAT	0.72	1.07	2.21	4.73	0.89	0.47	0.50	0.459	2
PERSIANN_CDR	0.71	1.07	2.30	4.85	0.90	0.46	0.51	0.556	3
RFE2	0.66	1.09	2.33	5.15	0.88	0.46	0.51	0.701	4
ARC2	0.61	1.11	2.43	5.58	0.86	0.47	0.49	0.791	5

### Spatiotemporal assessment of droughts via CHIRPSV2.0 precipitation estimates

#### Temporal patterns of drought characterization in the basin

**Fig. 5:**
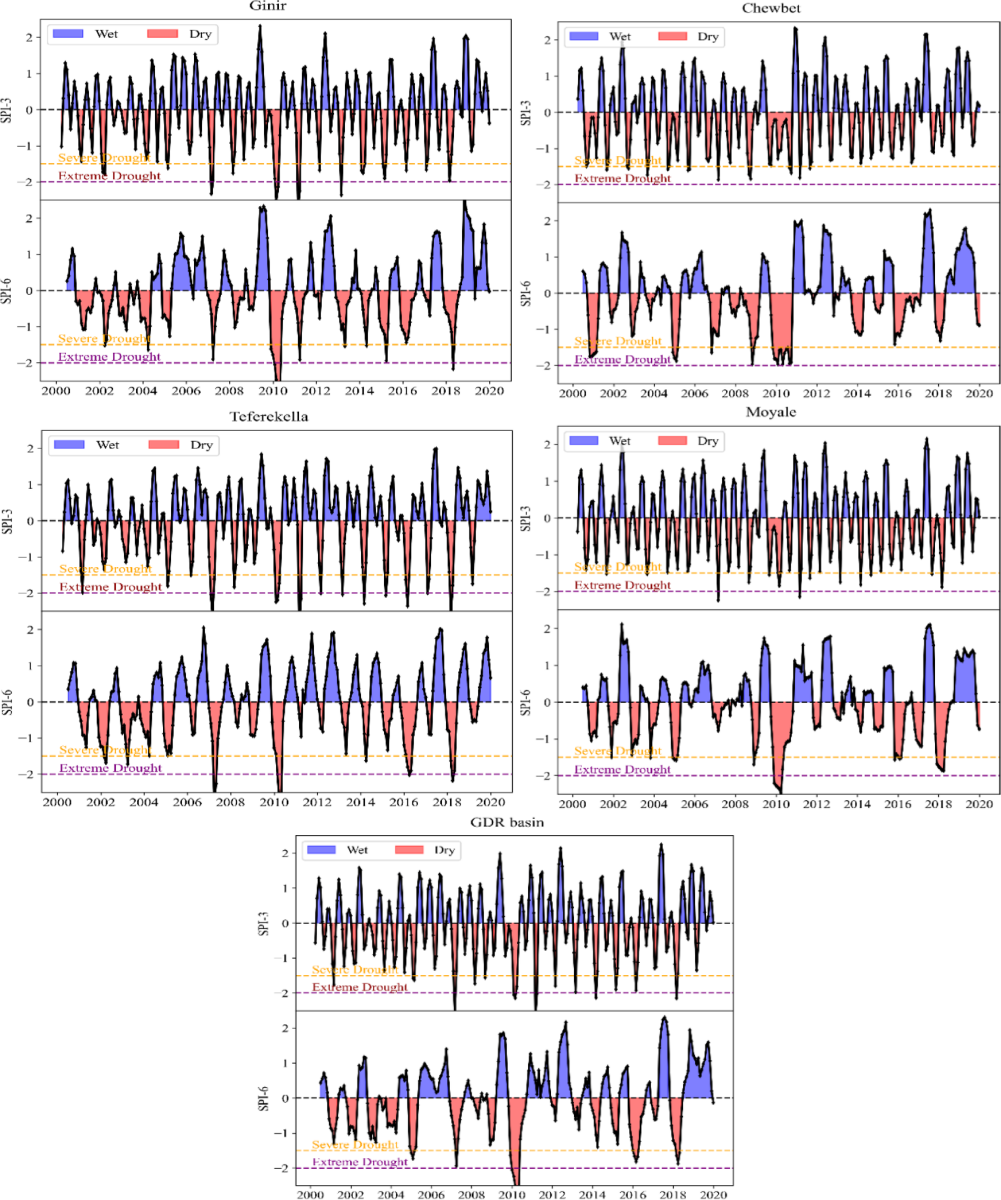
Temporal variation of the 3-month and 6-month Standardized Precipitation Index at four selected stations and the entire GDR basin during the period 2000–2020. Drought severity is classified based on SPI thresholds, with severe drought defined as SPI < − 1.5 and extreme drought as SPI < − 2.0, indicated by orange and purple dashed horizontal lines, respectively.

This study employed the 3-month (SPI-3) and 6-month (SPI-6) Standardized Precipitation Index to analyze meteorological drought from 1986 to 2020 using the best-performing CHIRPSV2.0 precipitation dataset. These indices assess precipitation anomalies over 3- and 6-month periods relative to historical averages. Figure 5 illustrates the temporal variations of SPI-3 and SPI-6 from 2000 to 2020 for four selected stations and the entire GDRB basin. Positive SPI values (blue) represent wet conditions, while negative values (red) indicate dry periods. The results of the SPI showed that all stations experienced drought in the same years, however, the severity of the drought varies by the station.

Based on the results shown in Fig. 5, the Ginir station experienced extreme droughts during the years 1994, 2008, 2011, 2012, and 2014 for SPI-3, and in 1994, 1997, 2000, 2010, and 2011 for SPI-6. In addition, the annual SPI-3 series in Teferekella station revealed that extreme drought was experienced in 1994, 1999, 2000, 2008, 2011, 2012, 2014, 2017, and 2019 whereas extreme drought was observed in 1988, 1992, 1997, 2000, 2008, 2011, 2017, and 2019 for SPI-6. Similarly, at the Moyale station, extreme drought events were recorded in 1990, 1991, 1992, 2010, and 2011 for SPI-12. Conversely, while no extreme drought events were observed at the Moyale and Chewbet stations for SPI-3, which are in a lowland area, mild to moderate drought conditions were recorded. However, Chewbet Station was exposed to extreme drought in 1999 for SPI-6. A high frequency of extreme drought events was experienced at the Teferekella and Ginir stations, which are in highland regions, compared to other stations. The highest frequencies of extreme droughts were observed at Teferekella and Ginir stations, which are situated in the highland regions of the basin.

Across the GDRB, SPI-3 reveals frequent short-term wet and dry fluctuations, with notable droughts around 2001–2003, 2009, 2010, 2015, 2016 across the GDRB. In contrast, SPI-6 captures more prolonged drought conditions, especially during 2001–2003 and 2014–2016, with some episodes reaching extreme severity. The most intense drought years were marked by severe water shortages and significantly below-average precipitation, reflecting substantial climatic stress across the region. These findings highlight high variability in precipitation patterns, where SPI-3 reflects short-term anomalies and SPI-6 indicates sustained drought conditions offering valuable insights for drought monitoring and long-term water resource planning.

#### Spatial pattern of drought characteristics in the basin

**Fig. 6:**
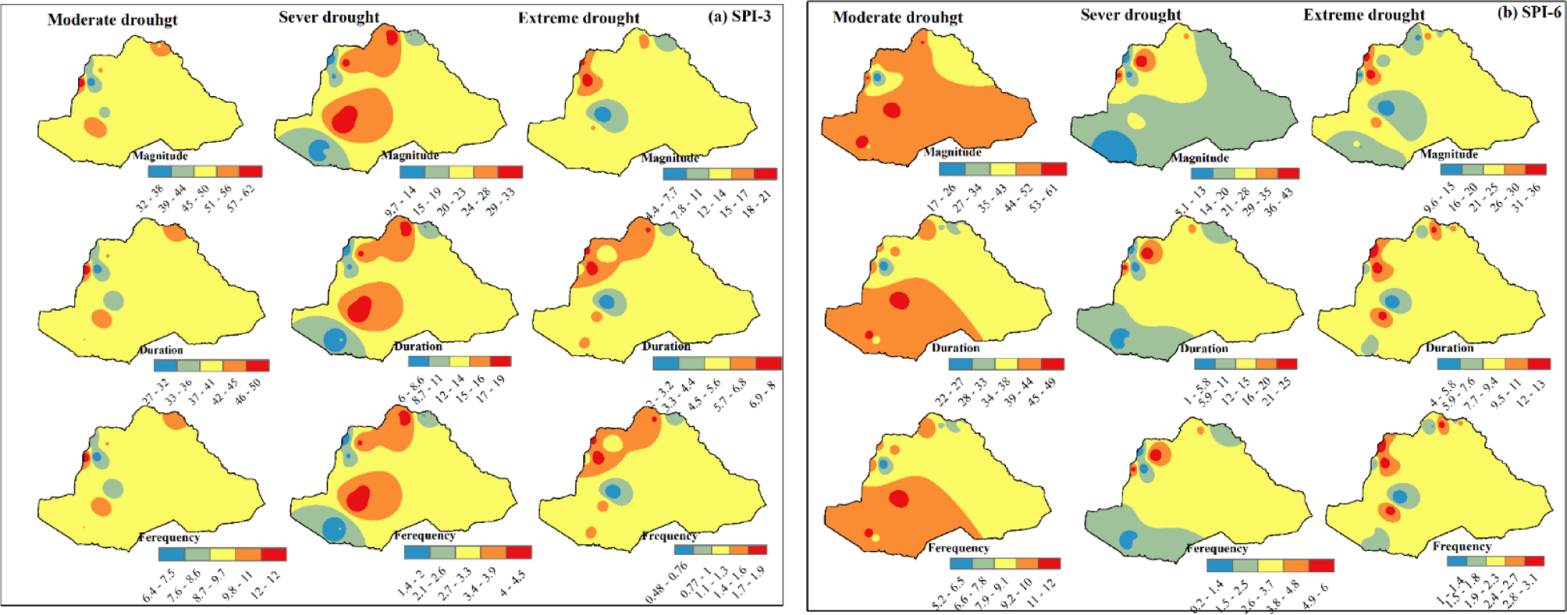
Spatial distribution of drought magnitude, duration, and frequency, for the 1986-2020 period using SPI-3 (**a**) and SPI-6 (**b**).

The drought characterization such as magnitude, duration, and frequency of the basin was analyzed using SPI-3 (a) and SPI-6 (b) at an annual time scale in the GDRB (Fig. 6). The results demonstrate that the spatial distribution of drought magnitude, duration, and frequency in the GDRB reveals different regions experience varying levels of vulnerability to droughts. The first row of the figures represents the magnitude, the second row represents the duration, and the third row represents the frequency of moderate, severe, and extreme drought events. As shown in Fig. 6a, SPI-3 indicates the highest magnitudes of moderate droughts in the northern and parts of the northwest. Severe droughts were more intense in the northern and south-central highlands, while extreme droughts occurred in isolated areas of the northwest. The frequency and duration of drought events also exhibit a similar distribution pattern to that of the magnitude. This distribution indicates that the northern and southcentral regions are particularly vulnerable to droughts, which significantly affect agricultural activities, reduce water availability, and lead to increased water stress. These regions face increased risks of crop failure, livestock losses, and long-term disruptions to water resources, highlighting the need for targeted interventions to mitigate the negative impacts of drought on both food security and water management.

The analysis of drought characteristics in the GDRB, based on the SPI-6, reveals remarkable spatial variations in drought magnitude, duration, and frequency across the region (Fig. 6b). Moderate droughts show a more widespread impact, with higher magnitudes, durations, and frequencies observed across the basin. These droughts, although less severe in terms of water deficit, occur more frequently and affect all regions of the GDRB. The consistent recurrence of moderate droughts highlights the cumulative stress on agricultural systems, water resources, and ecosystems, making long-term adaptation strategies essential to sustain livelihoods and mitigate water scarcity. The widespread nature of these droughts underscores the need for basin-wide water management practices to address the recurring challenges of moderate drought.

In contrast, severe droughts are more localized, affecting mainly the northern and central regions of the basin. These areas experience high magnitude, frequency, and prolonged duration of severe droughts, with significant impacts on agricultural productivity and water availability. The recurrence of severe droughts, particularly in the northern and central GDRB, poses significant challenges as the short recovery periods between drought cycles prevent the effective regeneration of water resources and agricultural systems. This pattern of severe drought emphasizes the urgent need for localized mitigation measures, such as rainwater harvesting, water conservation techniques, reforestation, afforestation, and climate-smart infrastructure, to manage the intensity and frequency of drought impacts in these vulnerable regions.

Extreme droughts, although less frequent, have the most intense drought characteristics, with the northern GDRB facing prolonged and severe impacts. The northern and some southern areas experience extreme water shortages during these events, which significantly disrupt agricultural activities and lead to water stress for both human and ecological systems. Although extreme droughts are less frequent, their severity causes long-term damage to water resources, so it is essential to implement preparedness strategies, including water storage systems and contingency plans, to deal with the occasional but highly destructive nature of these droughts.

### Association of drought with climate drivers: AMO, PDO, ENSO, and NAO

**Fig. 7:**
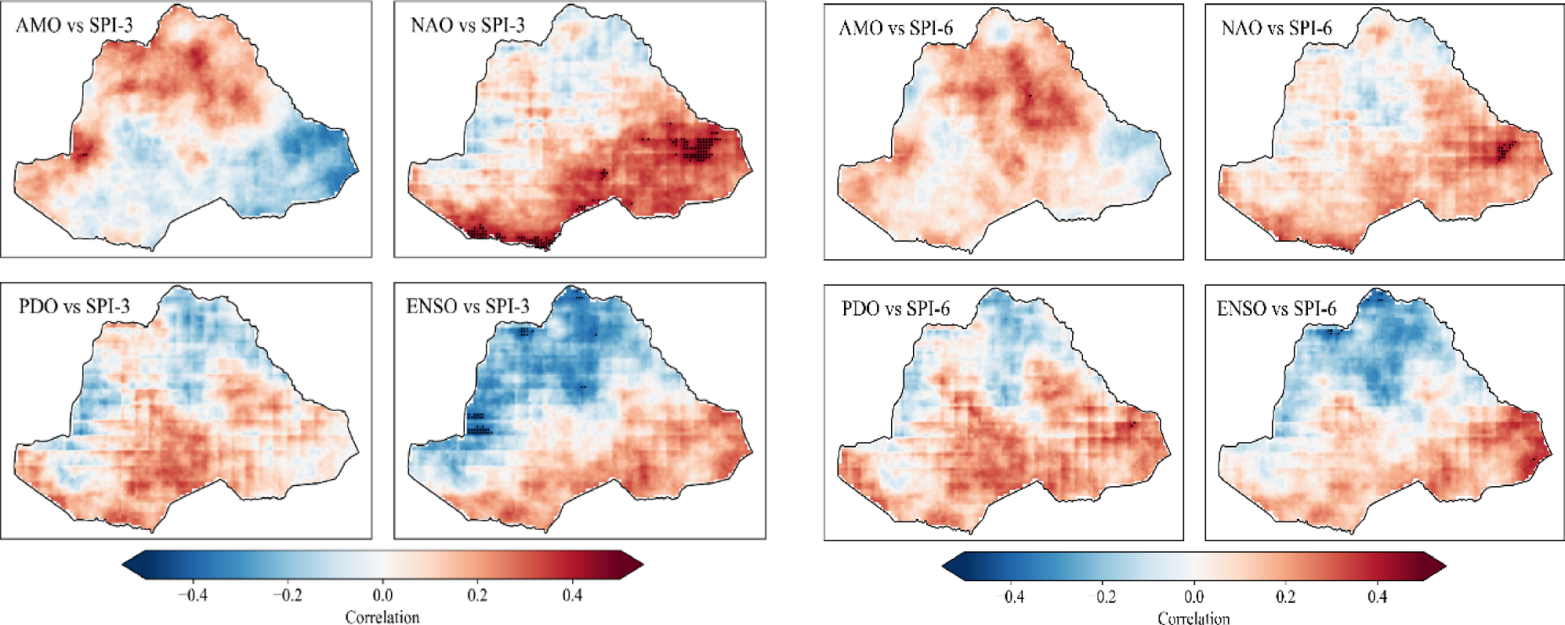
Association between drought and climate drivers over the study period.

The spatial correlations between drought indices such as SPI-3 (a) and SPI-6 (b) with the various climate drivers, including AMO, NAO, PDO, and ENSO, across the basin is presented in Fig. 7. The Pearson correlation method was applied to assess the relationship between drought indices and global climate indices. The results reveal that the AMO index is associated with a significant positive correlation (ranging from 0.2 to 0.4) with SPI-3 and SPI-6 in the northern and western regions, indicating that the positive phase of AMO is associated with wetter conditions likely due to enhanced moisture transport from the Atlantic, while negative correlations (ranging from − 0.2 to −0.4) are observed in the eastern region. however, the correlation of SPI-6 between AMO over the southern regions showed an insignificant positive correlation. This suggests that AMO influences seasonal precipitation patterns differently across the basin. This might be related to shifts in regional atmospheric circulation patterns^56–58^.

In contrast, the NAO oscillation showed a positive and significant correlation in the southern regions of the basin, suggesting that a positive NAO phase is linked to wetter conditions in these areas or that the regions tend to experience increased precipitation. Similarly, the PDO exhibits positive correlations in the southern region and negative correlations in the north, suggesting regional variations in drought response to PDO phases. The ENSO oscillations showed strong negative correlations in the northern part of the basin, suggesting that El Niño events are associated with drier conditions, whereas the southern region experiences positive correlations, linking El Niño to wetter conditions during the La Niña phases. These findings highlight the spatial variability in the influence of different climate drivers on precipitation patterns within the basin. As shown in Fig. 7, AMO, NAO, and ENSO exhibit stronger correlations with drought than PDO, suggesting that drought in the study area is only slightly influenced by PDO.

Table 7 shows that the climate indices have a moderate positive relationship with the 3-month SPI-3 in the GDRB (*R* = 0.6), with about 40% of the variation in SPI-3 explained by the combined influence of these indices (R² = 0.4). Among them, ENSO had a moderate positive effect (0.4), indicating that wetter conditions tend to occur during stronger ENSO phases. In contrast, PDO showed a moderate negative effect (−0.3), suggesting that it suppresses short-term rainfall. The roles of NAO (0.1) and AMO (−0.1) were comparatively weak. Overall, ENSO and PDO emerged as the main large-scale drivers of short-term precipitation variability in the basin.

For the 6-month SPI-6, the overall correlation was similar (*R* = 0.6; R² = 0.4), but the relationships were stronger. ENSO (0.9) and AMO (1.3) showed strong positive associations with SPI-6, while PDO (−0.5) remained a strong negative influence, and NAO (0.4) had a moderate positive effect. These findings suggest that large-scale ocean-atmosphere patterns play a greater role in shaping SPI-6 rainfall variability than SPI-3 in the GDRB, with AMO and ENSO driving wetter periods and PDO tending to suppress them.

**Table 7:** Correlation and regression statistics between standardized precipitation indices (SPI-3 and SPI-6) and large-scale climate indices in the GDRB.

Independent	Dependents	Regression coefficients	Multiple Regression (*R*)	Coefficient of determination (*R*^2^)
SPI-3	AMO	−0.1	0.6	0.4
	NAO	0.1		
	PDO	−0.3		
	ENSO	0.4		
SPI-6	AMO	1.3	0.6	0.4
	NAO	0.4		
	PDO	−0.5		
	ENSO	0.9		

## Discussions

### Performance evaluation

The statistical performance results showed that all the satellite rainfall products captured the spatiotemporal variation in rainfall in the GDRB. However, CHIRPSV2.0 and TAMSAT outperformed other satellite rainfall products at dekadal and monthly timescales, while TAMSATv3.1 and PERSIANN-CDR showed superior performance at daily or shorter timescales. This suggests that different satellite precipitation products are optimized for different temporal resolutions. CHIRPSV2.0 and TAMSAT perform better over longer time scales, such as decadal and monthly, probably because their algorithms are designed to capture broader rainfall trends and smooth out short-term variability. On the other hand, TAMSATv3.1 and PERSIANN-CDR excel at capturing more detailed, high-frequency rainfall events, making them more suitable for daily or short-term monitoring. This distinction highlights the importance of selecting satellite products based on the specific time scale of the analysis, as certain products are more reliable for long-term trend analysis, while others are better suited for short-term rainfall events. The observed performance differences between high and low-elevation regions are consistent with findings from previous studies, which also highlight the challenges of accurate rainfall estimation in diverse topographic environments. In this context, the satellite data performed better in Teferekella (Highland) compared to Moyale (Lowland), further illustrating how elevation influences rainfall accuracy. These examples emphasize the need for tailored approaches in rainfall estimation, accounting for varying topographic conditions. Several studies, such as^20,59^ have shown that satellite products tend to perform better in high-altitude regions due to well-defined orographic rainfall patterns, which are easier for satellite sensors to detect. In contrast, lower elevation regions, where convective rainfall is more common, often experience greater variability in rainfall detection accuracy. This challenge has been noted in studies such as^60,61^, highlighting the difficulty of capturing localized and rapidly evolving storms in lowland areas using satellite data. These factors explain why only a few products, such as PERSIANN-CDR, performed well in lowland areas in the present study.

The time scale of the analysis also influences the performance of satellite precipitation products. Numerous studies have shown that CHIRPSV2.0 performs exceptionally well at longer time scales, such as monthly and decadal, due to its integration of satellite data and ground station observations, which allows it to capture broader rainfall trends^62–64^. This supports the finding that CHIRPSV2.0 outperformed other products at these time scales, providing more reliable estimates for climate and hydrological studies. These results reinforce the need to select satellite products based on both the geographic characteristics of the region and the time scale of the analysis. Future improvements in satellite algorithms, especially for low-elevation regions, could help close the performance gap and improve the accuracy of precipitation estimates in different environments.

### Drought and its association with climate indices

The drought condition and its distribution pattern have been assessed in the GDRB for 1986 to 2020. As shown in the resulting Fig. 5 the temporal pattern of both the SPI-3 and SPI-6-time month scales showed that severe to moderate drought events were experienced in the study area for all stations, however, the severity of the drought varied in the station. For example, extreme drought was observed in 1988, 1991/1992, 1994, 1999/2000, 2008, 2011, and 2012 for both SPI-3 and SPI-6-time scales. The highest extreme drought years were marked by severe water shortages and below-average precipitation, reflecting significant climatic stress and persistent drought conditions. Indeed, these years were among the worst drought years in the history of Ethiopia. Several studies^65–68^ have highlighted that severe droughts occurred during these years, resulting in significant losses to both lives and the economy. Additionally, the study^53^, identified multiple drought years including 1984, 1992, and 2011, with a high frequency of moderate to extreme drought conditions in the Borena area. Moreover, these results are in line with^69^, who reported significant drought events were recorded in 1991/1992, 1998/1999, and 2010/2011, with the latter being particularly severe in southern Ethiopia. Additionally, a drought characteristics study in Eastern Ethiopia revealed that severe drought events occurred most frequently in the basin with a general occurrence of drought events every two years across all stations^70^.

Furthermore, the spatial distribution of drought characteristics in the GDRB has been investigated using SPI-3 and SPI-6, the results reveal different regions experienced varying levels of drought vulnerability in the study area. As shown in Fig. 6, drought characteristics indicate varying levels of drought severity across spatial scales for both SPI-3 and SPI-6 time-month scales. The basin experienced various drought events, ranging from moderate to extreme for both SPI-3 and SPI-6. Severe and extreme drought events, characterized by their magnitude, duration, and frequency, were particularly evident in the northern western and central parts of the basin for SPI-3. Similarly, these drought characteristics were also observed in the same regions for SPI-6, though with a increased spatial extent. This indicates that while severe and extreme drought conditions are consistently present in the northern and central parts of the basin, their spatial extent boosts as the time scale of the SPI increases (from SPI-3 to SPI-6). This suggests that shorter-term droughts (SPI-3) are more localized, while longer-term droughts (SPI-6) cover a larger area. This indicates that shorter-term droughts may have a more intense localized impact, whereas longer-term droughts, though possibly less intense, can influence a broader region, posing different challenges for resource availability and drought management strategies across time scales. This study is in line with^70^, who reported that moderate to extreme drought events occur approximately once every two years across the basin, however, severe and extreme drought events are less frequent, occurring nearly once every three years.

A review of these results indicates that the influence of different climate drivers on drought conditions within the basin varies according to regional characteristics. The correlation patterns demonstrate how distinct climate oscillations influence precipitation variability across different regions within the study area. For instance, the AMO’s impact, as evidenced by positive correlations with SPI-3 and SPI-6 in the northern and the western regions, suggests that its positive phase may facilitate moisture transport, particularly from the Atlantic, leading to wetter conditions. This result aligns with the findings of^71,72^, who reported that the dynamics of moisture transport merit further investigation, implying a connection between AMO phases and shifts in atmospheric circulation patterns over the basin.

It is noteworthy that the NAO also exhibits a pronounced positive correlation in the southern regions, indicating that its positive phase is conducive to precipitation in those areas. This could indicate an interaction with local atmospheric factors, where the positive NAO phase aligns with patterns conducive to moisture availability in the south^73^. Furthermore, the PDO’s positive correlations in the south but negative in the north suggest a varied regional response within the basin, hinting at a more complex relationship with seasonal precipitation that might not be as influential as other indices but still shows significant spatial variation.

The contrasting correlations of the ENSO in the northern and southern regions align with the known precipitation anomalies that occur during El Niño and La Niña events. As reported by^74^ the El Niño events may result in drier conditions in the northern region, potentially due to changes in atmospheric moisture levels or enhanced anticyclonic activities. Conversely, the La Niña phases are associated with enhanced rainfall in the southern region, as highlighted by the findings of^75^. These observations provide insights into the influence of each climate driver on seasonal precipitation and potentially intraseasonal precipitation, emphasizing the necessity to consider the temporal dynamics of these oscillations.

The spatial variability observed in the correlations between the PDO, NAO, and ENSO suggests that while the PDO exerts a relatively limited impact, the AMO, NAO, and ENSO exert a stronger influence on drought conditions. These results align with the findings of^76^, who reported that the variability observed among climate drivers is likely attributed to their ability to influence moisture-laden winds and alter precipitation patterns. These results emphasize that drought responses across the basin are not uniform but instead are modulated by regional climate dynamics and their interaction with large-scale oscillations. This underscores the importance of region-specific climate adaptation strategies.

## Conclusions

In this study, we evaluated the performance of multiple satellite rainfall products (CHIRPSV2.0, RFE 2.0, TAMSAT V3.0, PERSIANN-CDR, and ARC2), analyzed drought characteristics and their linkages with climate indices in the Gelana Dawa River Basin from 1986 to 2020. The performance of satellite rainfall estimates was assessed through both categorical and continuous statistical analyses. CHIRPSV2.0, identified as the best-performing product, was used to analyze drought conditions using the Standardized Precipitation Index to track drought events. Finally, the multiscale teleconnection between SPI-based drought indices and various climate indices was examined using the Pearson correlation coefficient.

The study demonstrates better performance of CHIRPSV2.0 with strong r and lower RMSE, ME, and bias, at monthly time scales, indicating its suitability for drought monitoring in the region. Therefore CHIRPSv2.0 is the most suitable satellite product to assess the spatiotemporal meteorological drought characterizations for 3- and 6-month periods across the basin. The study indicates that the area has experienced extreme to moderate drought conditions from 1986 to 2020, with the worst droughts occurring in 1988, 1991/1992, 1994, 1999/2000, 2008, 2011, and 2012. The results indicate that the northern and central western regions are particularly vulnerable to drought, significantly affecting water and agricultural resources. As a result, these areas should be prioritized for targeted water management strategies and agricultural adaptation measures to mitigate the impacts of future drought events.

The correlation between drought and climate indices reveals a highly spatially variable impact of global climate indices on precipitation in the GDRB. The AMO and NAO predominantly exhibit positive correlations in the northern and central regions, potentially contributing to wetter conditions, while the PDO and ENSO generally demonstrate negative correlations, particularly influencing the southern, central, and eastern parts of the basin. This spatial variability underscores the necessity of regional climate modeling and the development of local adaptive strategies to effectively address the diverse impacts of global climate variability. Ultimately, a tailored approach is essential for enhancing resilience to drought and optimizing water resource management in the region.

## Data Availability

The datasets used during the current study are available from the corresponding author upon reasonable request.
